# An assessment of the perceptual elements of urban streets based on the context of urban tourism - The case of Sheffield

**DOI:** 10.3389/fpubh.2024.1380723

**Published:** 2024-04-09

**Authors:** Siqi Huang

**Affiliations:** School of Art and Design, Sheffield Hallam University, Sheffield, United Kingdom

**Keywords:** locals, travelers, IPA-Kano model, city streets, evaluation of perceptual elements

## Abstract

**Background:**

After COVID-19, more and more travelers are more inclined to walk in cities, and the sensory elements of streets can have a significant impact on urban tourism. Local residents and travelers have different perceptions of the street and preferences for its use. The purpose of this study is to evaluate and analyse the streets from the perspective of locals and travelers.

**Method:**

In this study, a questionnaire was designed to obtain local residents' and travelers' evaluations of the sensory elements of the street and a quadrant analysis of the street's sensory elements was carried out using the IPA-Kano model.

**Results:**

The results of the study show that travelers are particularly concerned about maps and signage guidance, while local residents are more concerned about the green environment of the surroundings and how well it is maintained.

**Conclusion:**

There is a difference in the indicators chosen by the two groups in the results of the comparison between locals and travelers, and this study is hoped to provide some data support for future urban managers and designers to learn from and refer to for street improvements and renewal.

## 1 Introduction

Tourism is one of the fastest-growing industries in the world. The industry is known for creating jobs and helping to reduce unemployment and poverty (1–4). Today, tourism is of unparalleled importance in tourist regions, and tourism largely underpins urban economic stability and recovery, most notably in the aftermath of the COVID-19 pandemic. During COVID-19, people traveled less and the world's tourism industry suffered from an unprecedented downturn. With the later stages of COVID-19, tourism has caused cities to rise economically, so governments are placing great emphasis and support on restoring tourism, while safeguarding public health, as well as employment (5–8). In 2022 there was a surprising rise in the growth rate of global tourist arrivals, with tourist arrivals in the Middle East climbing to 83% of pre-COVID-19 levels according to the United Nations, Europe reaching nearly 80% of pre-COVID-19 levels, and both Africa and the Americas regaining 65% of their pre-COVID-19 levels of tourist arrivals. The importance of tourism to the economic stability and recovery of major countries in the aftermath of the epidemic has been demonstrated in practice and data.

After the epidemic and in the context of the tourism outbreak, people were eager to travel and visit the city, and the image of the city in all its forms played a more important role in people's overall perception than before the epidemic. Urban public space consists mainly of streets (9). Public spaces such as parks, playgrounds and shopping malls in some European cities are built on streets. Streets connect various amenities as well as scenes, so local residents or travelers spend a lot of time on city streets (10). Streets are also important platforms to showcase the city. Therefore, the importance of streets becomes apparent; from the traveler's perspective, city streets are the first public spaces that tourists see when they enter the city, and they are the first impression that tourists have of the city. In the context of urban tourism, urban walking has become the subject of research (11). Tourists walk in cities more to explore the unknown, improve the quality of the tourist experience, seek authenticity, and build place attachment (12, 13). In the era of self-media everywhere, the phenomenon of urban walking continues to appear on online platforms, and the combination of urban walking and self-media makes a lot of the city's image not only known through offline travel, but also through videos and photos posted by travelers to allow more people to experience online travel, attracting more attention to different cities, thus driving the attractiveness of tourism. After getting a lot of attention, city planners need to fully consider the needs of tourists, who do not have the same spatial familiarity as residents (14). Therefore, the perceptions of street space, design elements and amenities have a huge impact on travel, which greatly reflects the image of the city and its sustainability for tourism development.

Many countries around the world, including China, are looking at the symbiosis between the city and the traveler, maintaining the original environment as much as possible while enhancing the travel experience. With the amount of visual space available in the streets, it is crucial and necessary to determine which elements are more attractive to travelers. This study intends to assess the street by travelers and then determine through IPA-Kano which elements are the most attractive to them, which elements they find less satisfying and which elements should be optimized appropriately. The aim is to analyze the data from the IPA-Kano model of travelers' perceptions of the elements of the street and to produce results that will inform the government.

## 2 Literature review

### 2.1 Street assessment refers to relevant research

Existing research has focused on the safety, comfort and level of maintenance of the space and associated facilities.

For the phenomenon of security, Painter (15) found that public lighting was a very important factor in security by improving public lighting facilities in three streets in the UK and found that pedestrians in the streets increased significantly after the public lighting was improved (15). Navarrete-Hernandez et al. (16) evaluated the security of public spaces and they concluded that public lighting as well as surveillance was a very important factor in security. Mohammed et al. (17) evaluated public spaces in Hail, Saudi Arabia, and they concluded that nighttime lighting and floor tiles are security factors. Lorenzo et al. (18) analyzed and categorized the quality of public spaces, and among the security indicators they identified security surveillance, lighting facilities, and non-slip floor tiles as necessary factors. Sadeghi et al. (19) investigated women's preferences in public spaces, and they emphasized that lighting, directional signage, surveillance, and so on are important factors in creating a sense of security in urban spaces (19). Au-Yong et al. (20) a prioritization of facilities in Malaysian public spaces from the perspective of tourists and they found that safe public spaces are preferred by tourists, with lighting and signage guidance being the highest priority security factors (20). Subramanian et al. (21) evaluated urban recreational public spaces for older adults and their findings proved that aspects such as street lighting, security guards, maps, signage, handrails, and ramps are more important for older adults

For the comfort phenomenon, women go to public spaces with greenery more than men (22). For women, children or the elderly, public spaces need benches to rest on and unpolluted clean urban spaces can be more relaxing. The appropriate width of the street space makes it more comfortable and convenient for women to walk or push prams (19). Designing appropriate seating, increasing the number of high-quality water elements, and increasing the number of green spaces will allow residents and visitors to enjoy outdoor spaces (17). Cleaning and maintenance the abundance of plant species and a sufficient number of benches can allow visitors and residents to enjoy better visuals and comfort (23). In Rose and Basri's (20) study, they demonstrated that poor visitor satisfaction is due to poorly maintained washrooms, especially a lack of cleaning. From a visitor's perspective, bins are a high-priority facility (20). Mohamad Muslim et al. (24) emphasized that park visitors are likely to be attracted by natural beauty. Anuar and Muhamadan (25) argue that the condition of benches and tables in public spaces should be maintained to accommodate passive recreational activities.

Pleasurability is a factor that measures feelings about the environment and is an individual's perception of happiness and satisfaction with an environment (26). Some have noted that perceived pleasure increases a traveler's willingness to share the experience with others (27). For most travelers, the choice of destination is a crucial matter, as it is the basis for their subsequent actions (28). Travelers seek advice from online social networks when planning a trip (29). An example of this is the popular website TripAdvisor, where nearly 200 million travelers search for information and references about destinations, restaurants, and hotels that influence their travel plans (30). This also shows that travelers tend to look for places they want to visit on the internet before traveling, making traveling more enjoyable and interesting, as in Table 1.

**Table 1:** Literature compilation of public space indicators.

**References**	**Evaluation indicators**	**Classification of indicators**
Painter (15)	Public lighting	Safety
Navarrete-Hernandez et al. (16)	Public Lighting, Surveillance	
Mashary Alnaim et al. (17)	Night lighting, floor tiles	
Lorenzo et al. (18)	Security monitoring, lighting, non-slip floor tiles	
Sadeghi et al. (19)	Lighting, guide signs, surveillance	
Au-Yong et al. (20)	Lighting, signage guidelines	
Subramanian et al. (21)	Lighting, Security, Maps & Signage, Handrails, Ramps	
Ode Sang et al. (22)	Green plant richness	Comfortableness
Sadeghi et al. (19)	Benches, street cleanliness, street aspect ratios, maintenance tables and chairs	
Mashary Alnaim et al. (17)	Seats, water elements, green areas	
Madureira et al. (23)	Cleanliness, Richness of plant species Benches	
Rose et al. (31)	Toilet maintenance	
Au-Yong et al. (20)	Maintaining the bins and cleaning, Netflix hit the ground running	
Mohamad Muslim et al. (24)	Maintenance of greenery	
Anuar et al. (25)	Maintenance of benches and tables	
Intason et al. (32)	Regular cultural and business events	Cheerfulness
Zhou et al. (33)	Network Recommended Locations	

### 2.2 IPA-Kano

IPA-Kano solves the shortcomings of IPA method and KANO, IPA-Kano makes the research results more accurate and intuitive.

In the service domain, Tseng (34) classified and diagnosed service attributes based on the IPA-Kano model, which provided managers with more valuable and accurate information to identify key drivers. Jiang et al.'s (35) study used the SERVQUAL model of service quality indicators, combining the fuzzy Kano model and IPA from the perspective of tourist satisfaction to establish a service quality evaluation system for recreational fisheries. Chen (36) proposed a customer-driven framework that integrates the strengths of the traditional IPA and the Kano model to assist in the planning of service strategies.

In the field of architectural design, Yin et al. (37) in examining Xi'an's residential satisfaction, the IPA-Kano model considers both implicit and explicit importance to differentiate between three types of factors that have different impacts on the formation of residential satisfaction. The combination of basic factors, key performance factors, excitement factors and their actual performance identifies development priorities for neighborhood self-improvement and priorities for inter-neighborhood competition. These priorities enable local governments to deploy scarce resources to effectively improve the residential satisfaction of existing residents and attract more residents from other neighborhoods (37).

## 3 Method

### 3.1 Introduction to the IPA-Kano model, method operation

**Figure 1:**
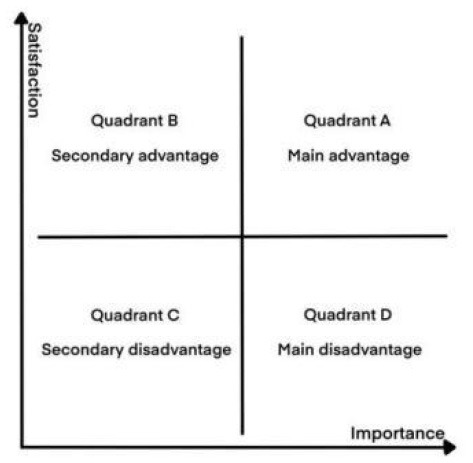
IPA model.

**Figure 2:**
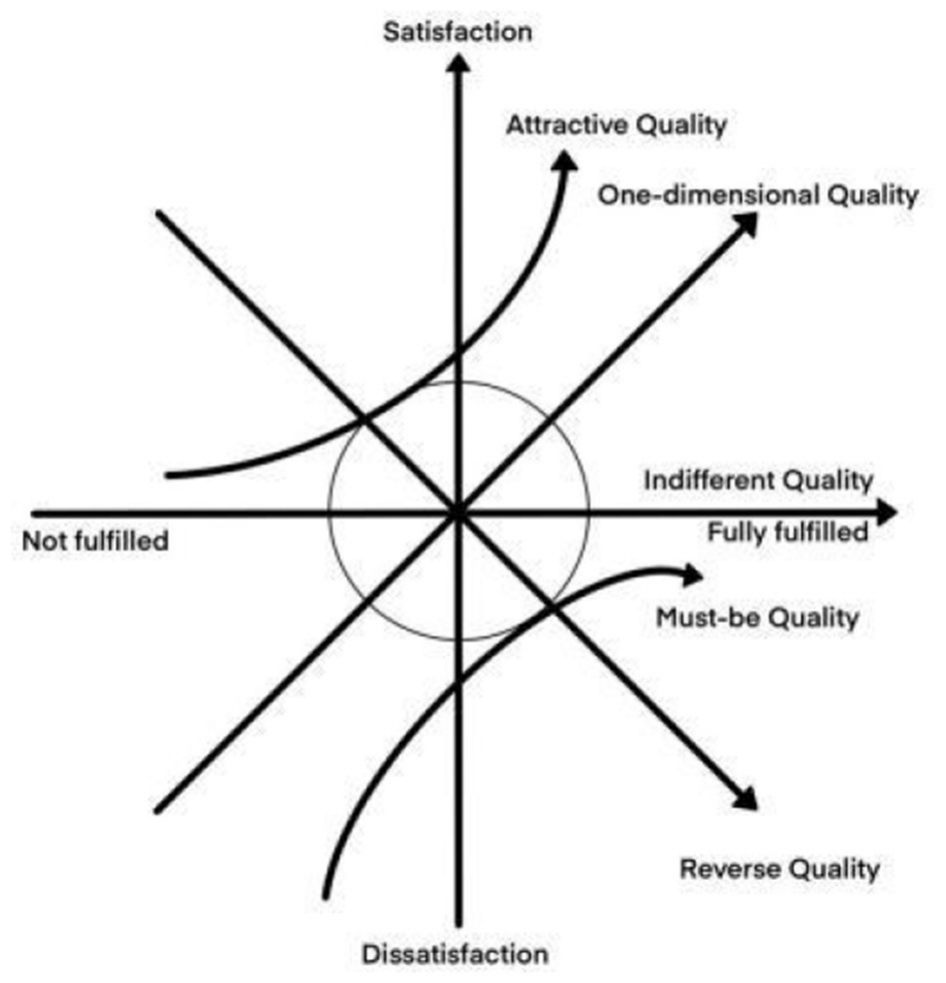
KANO model.

For the selection of methods to quantify spatial elements, the most commonly used are Importance-Performance Analysis (IPA) and Kano models Satisfaction degree of the measurement tool (38, 39). IPA was proposed by Martilla and James (39) to evaluate and prioritize factors of overall user performance. The satisfaction and importance of each IPA indicator are placed as coordinates on a four-quadrant graph, and the elements falling into different quadrants have different meanings (Figure 1). However, in practice, the relationship between elemental attribute performance and overall performance is non-linear and asymmetric (40). Therefore, this problem can be better solved by using the Kano model proposed by Japanese scholar Noriaki Kano, which divides user needs into five levels: attractive quality, one-dimensional quality, must-be quality, indifferent quality, and reverse quality. Elements under different need categories have different degrees of influence on overall performance (Figure 2), but the model itself cannot directly derive results and recommendations (38).

**Figure 3:**
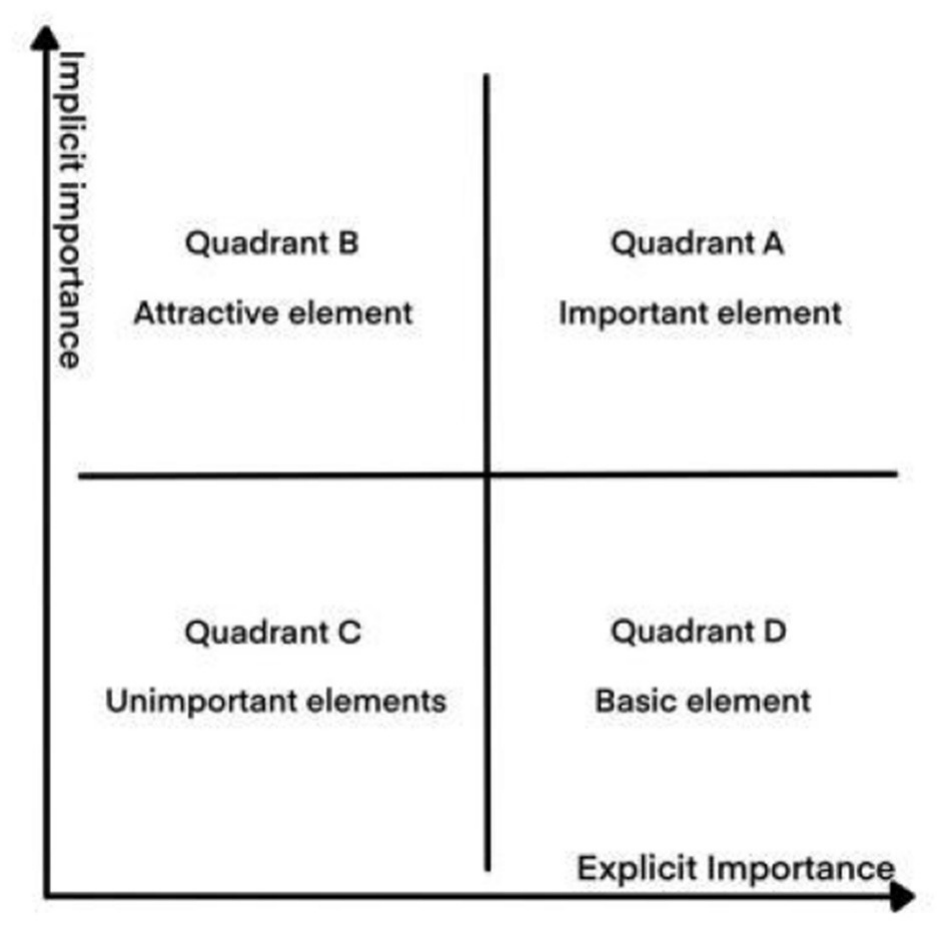
IPA-KANO model.

Matzler et al. (39) proposed the IPA-Kano model by combining the IPA model and the KANO model. It combines the advantages of IPA for judging the priority of relevant elements and Kano for evaluating the attributes of user requirements, with the horizontal coordinate indicating explicit importance and the vertical coordinate indicating implicit importance. Important elements in quadrant A have the highest explicit and implicit importance. Quadrant B are attractive elements with low explicit importance and high implicit importance. Quadrant C are unimportant elements with the lowest explicit and implicit importance. Quadrant D are basic elements with high explicit importance and low implicit importance (Figure 3). The model provides user needs prediction and causal path analysis, which guides the measurement of design elements and suggests improvement strategies (38).

The research in this project focuses on basic, important and attractive elements. The most important is the linear effect of the importance elements on overall satisfaction, if they are satisfied they increase overall satisfaction and vice versa. The basic elements do not affect overall satisfaction if they are satisfied, but if they are not satisfied, they decrease overall satisfaction. The remaining non-essential satisfaction elements do not affect overall satisfaction if they are satisfied and therefore do not require attention.

Although it is possible to derive evaluation results for the three street elements, this is not sufficient to provide an optimization path for their development. Since the contribution of the street elements to overall satisfaction depends on their actual satisfaction, the evaluation results should be combined with the satisfaction level in order to determine an optimization path for the street elements. It is important to note that the effects of basic and attractive elements on overall satisfaction are asymmetrical. For example, overall satisfaction is not improved when the basic element exceeds expectations but rather decreases if the basic element is not implemented. If the Attractiveness Element is not implemented, it does not lead to a decrease in overall satisfaction, but if the Attractiveness Element is implemented, it can increase overall satisfaction. The effect of the importance element on overall satisfaction is directly proportional to the degree of its implementation. Therefore, the order is basic elements, importance elements and attractiveness elements (38). The actual performance of street element satisfaction is divided into three categories (Table 2), reflecting different levels of performance. The top five are the best performers, the bottom five are the worst, and the others are relatively mediocre. We can derive an optimization path by constructing six priorities.

**Table 2:** Improvement priorities.

**Improvement priority**	**Quadrant**	**Satisfaction ranking**
1	Basic element	11-15
2	Important element	11-15
3	Attractive element	11-15
4	Basic element	6-10
5	Important element	6-10
6	Attractive element	6-10

### 3.2 Indicator system

**Figure 4:**
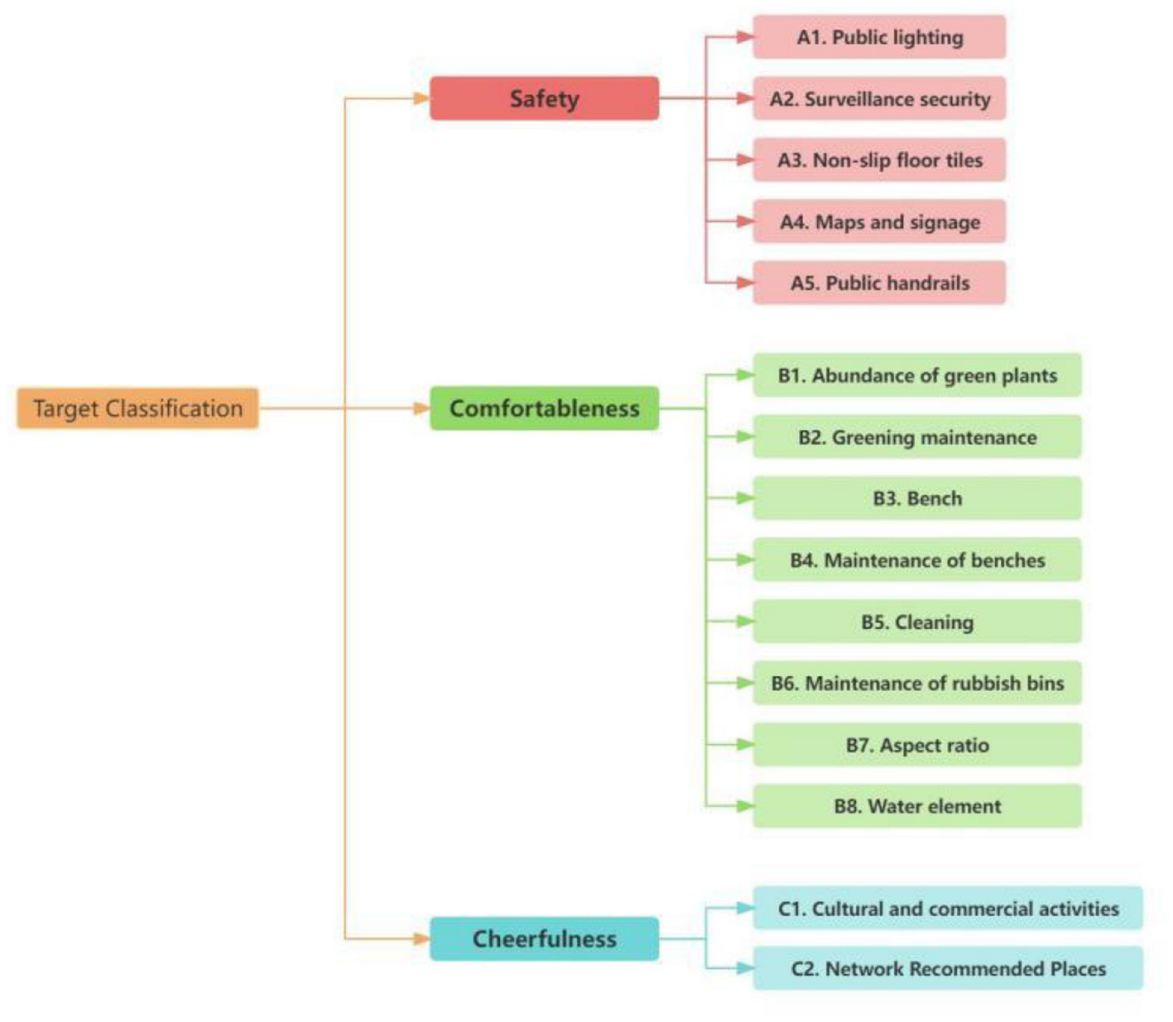
Street indicators.

Based on the literature, this study concludes that the perception of urban streets is divided into three main categories and 15 factors, which are safety, comfort and pleasure. The safety phenomenon includes five factors, which are A1. Public lighting, A2. Surveillance and security, A3. Non-slip floor tiles, A4. Maps and signs, and A5. Public handrails. Eight factors were included in the comfort phenomenon, namely B1. Abundance of greenery, B2. Maintenance of greenery, B3. Benches, B4. Maintenance of benches, B5. Cleanliness, B6. Maintenance of litter bins, B7. Aspect ratio and B8. Water elements. Two factors are included in the pleasantness phenomenon, C1. Cultural and commercial activities and C2. Places recommended by the internet (Figure 4).

### 3.3 Questionnaire design

The importance questionnaire was based on a 9-point Likert scale containing 15 questions based on local and traveler preferences for the three street factors. For example, on the safety factor public lighting, it asks “Do you think public lighting is important to West Street?” where “1” means very unimportant, “9” means very important, and “2” to “8” gradually increase the score as the level of importance increases. importance gradually increases. The weighted average score of each variable was used as the importance score to measure the apparent importance of each factor in the street. The satisfaction questionnaire was designed to allow users to rate their actual satisfaction with the three street factors on a nine-point Likert scale. For example, for the factor Public Lighting in Safety, it asks “How satisfied are you with the public lighting on the current West Street?” where “1” means very dissatisfied, “9” means very satisfied, and “2” through “8” increase the score as the level of satisfaction increases. The level of satisfaction gradually increases. The weighted mean score of each variable was used as the satisfaction score, reflecting the actual satisfaction of the street factor. The Importance and Satisfaction Questionnaire not only asked questions about the 15 factors, but also recorded the gender of the participants and whether the participating users were locals or travelers.

### 3.4 Experimental process

**Figure 5:**
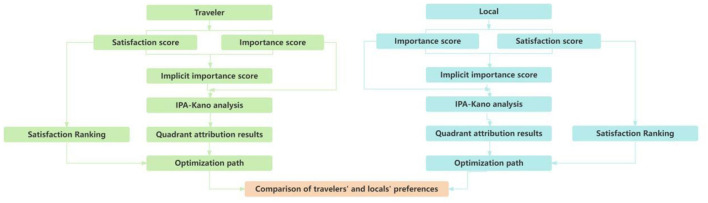
Experimental procedure diagram.

The experimental procedure consisted of the following five processes (Figure 5).

(1) The authors conducted and collated questionnaires to calculate the average importance and satisfaction of travelers and locals for each of the three street indicators.
(2) Bivariate correlation analyses of the respective importance and satisfaction of travelers and locals were conducted using spss27 statistical data analysis software to extract their corresponding implicit importance.

In this case, bivariate correlation analyses were conducted using SPSS 27.0 to extract the implied importance of the streets, which was calculated using the following Equation (1).


(1) $$r_{xy}=\frac{\sum xiyi-n\;xy}{\left(n-1\right)s_{x}s_{y}}=\frac{n\sum xiyi-\sum xiyi-\sum xi\sum yi}{\sqrt{n\sum x_{i}^{2}-}{\left(\sum x_{i}\right)}^{2}\sqrt{n\sum y_{i}^{2}}-{\left(\sum y_{i}\right)}^{2}}$$

The coefficients take values between −1.0 and 1.0, with variables close to 0 indicating no correlation and close to 1 or −1 indicating strong correlation.

(3) IPA-Kano analysis was used to present the results of the comparison between explicit and implicit importance, with the horizontal axis using importance data to represent explicit importance and the vertical axis using binary correlation analysis data of importance and satisfaction to describe implicit importance. The mean values of the 15 elements of explicit and implicit importance were used as the center coordinates, with each element corresponding to a quadrant plot.
(4) Analyze the optimization path based on the quadrant attribution results of the street elements, combined with the performance ranking of each element attribute.
(5) Based on the results of the above data, a comparative analysis of the elements of street perception by travelers and local residents was conducted to find their commonalities and differences.

### 3.5 Sheffield case presentation

**Figure 6:**
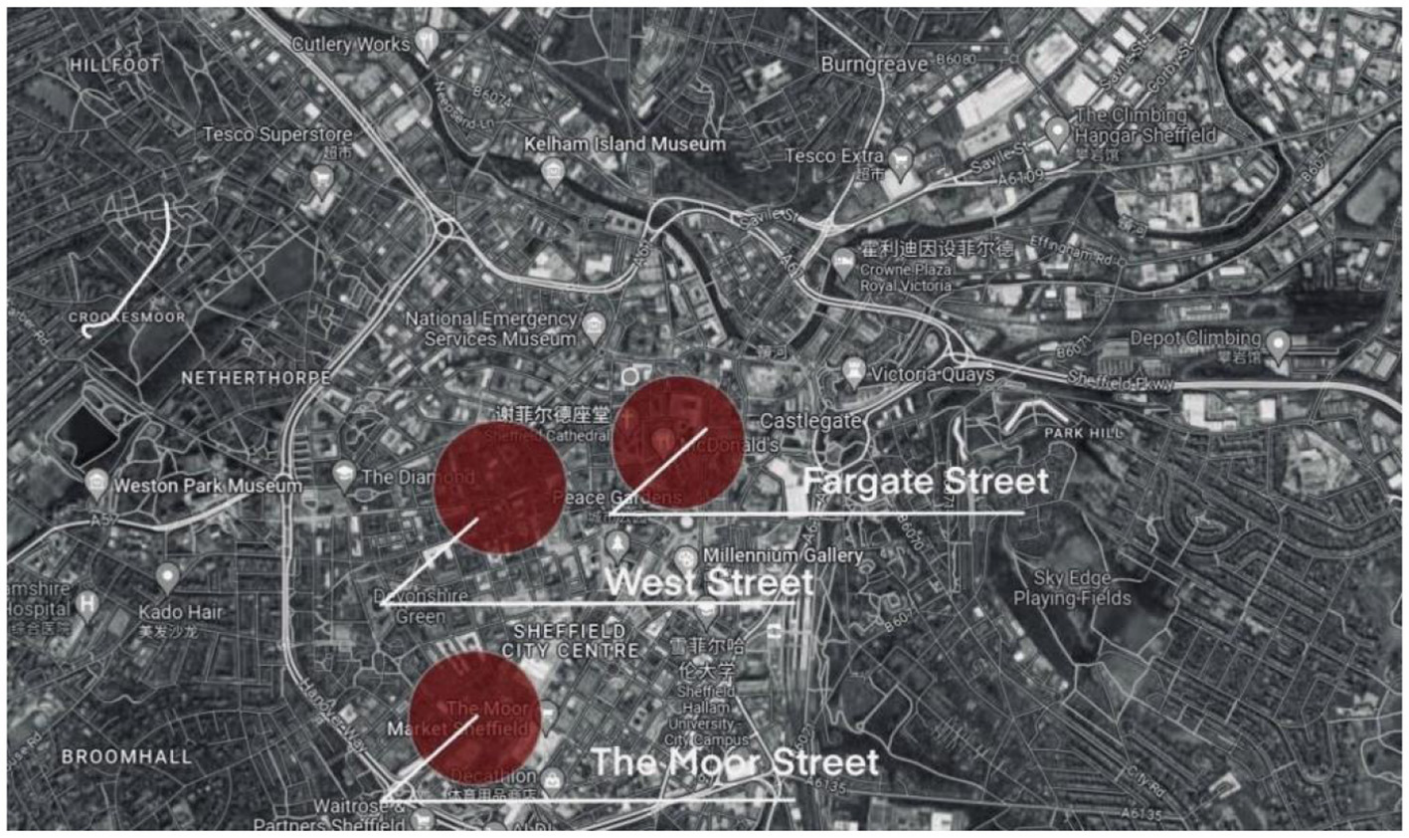
Three streets of Sheffield.

This study is based on three streets in Sheffield, England, namely West Street, Fargate Street and The Moor Street (Figure 6). All three streets are located in the city center of Sheffield, and West Street hosts a wide range of cultural and commercial events during holidays and weekends, so both locals and travelers are willing to participate in activities here. Fargate is a pedestrianized and shopping area in Sheffield, England, which often hosts commercial activities such as amusement rides, attracting a lot of locals to attend events here with their children. The Moor, a very famous shopping street in Sheffield city center, is more popular with travelers than locals, and hosts commercial events for food and entertainment at Christmas and Easter every year.

### 3.6 Participants

The participants in this study are local residents of Sheffield and travelers from other cities or countries. A total of 1,071 questionnaires were distributed in this study and 1,025 valid questionnaires were recovered after deleting the invalid questionnaires (Table 3), of which 360 questionnaires were distributed in Fargate Street and 343 valid questionnaires were recovered, 355 questionnaires were distributed in The Moor Street and 340 valid questionnaires were recovered, 356 questionnaires were distributed in West Street and 342 valid questionnaires were recovered. A total of 356 questionnaires were distributed on West Street and 342 valid questionnaires were returned. The questionnaires were distributed from November 2022 to September 2023, and were paid £0.30 per questionnaire through a variety of social apps online, and offline on the street where people were randomly approached to fill in the questionnaires. The 15 elements of the questionnaire were then analyzed for reliability and validity using SPSS 27 statistical software, and the Cronbach values were basically above 0.8, proving that the questionnaire had good reliability. The significance value was 0 and the validity was good.

**Table 3:** Gender and participant data of the questionnaire.

**Street name**	**Gender and participants**	**Quorum**	**Percent**
Fargate Street	Male	164	47.8
	Women	176	51.3
	Non-binary/third gender	2	0.6
	Prefer not to say	1	0.3
	Traveler	200	58.3
	Local	143	41.7
The moor street	Male	170	50
	Women	165	48.5
	Non-binary/third gender	3	0.9
	Prefer not to say	2	0.6
	Traveler	189	55.6
	Local	151	44.4
West street	Male	159	46.5
	Women	177	51.8
	Non-binary/third gender	4	1.2
	Prefer not to say	2	0.6
	Traveler	186	54.4
	Local	156	45.6

## 4 Results

### 4.1 Description of basic data

According to the values in Table 4, cleanliness is the most important indicator considered by both travelers and locals in Fargate Street, while public handrails are the least important in their opinion, and in the evaluation of satisfaction, height to width ratio is the indicator with the highest level of satisfaction, while the abundance of greenery is the indicator with the lowest level of satisfaction. Next, according to the values in Table 5, cleanliness is the indicator considered the most important in The Moor Street, while aspect ratio is the least important indicator in their evaluation, in the evaluation of satisfaction, locals are the most satisfied with aspect ratio, the richness of greenery is the least satisfied, travelers are the most satisfied with cultural and commercial activities, and public handrails, an indicator with the had the lowest level of satisfaction. Finally, according to the data in Table 6, locals and travelers consider cleanliness to be the most important indicator, the water element was rated as the least important indicator by locals, and the aspect ratio was rated as the least important indicator by travelers. Both locals and travelers rated the places recommended by the network as having the highest level of satisfaction and the lowest level of satisfaction with the abundance of green.

**Table 4:** Importance and satisfaction of Fargate street indicators.

**(Math.) factor**	**Fargate street**							
	**Local**				**Traveler**			
	**Importance**		**Satisfaction**		**Importance**		**Satisfaction**	
	**Mean**	**Ranking**	**Mean**	**Ranking**	**Mean**	**Ranking**	**Mean**	**Ranking**
A1	7.58	3	6.02	2	7.7	3	5.62	8
A2	7.42	4	5.45	9	7.22	4	5.51	11
A3	6.7	8	5.44	10	6.64	9	5.53	10
A4	6.62	10	5.79	5	6.72	8	5.82	5
A5	4.82	15	5.8	4	4.89	15	5.64	6
B1	6.66	9	4.64	15	6.55	10	4.97	15
B2	6.87	7	4.98	14	6.95	7	5.39	13
B3	6.92	6	5.47	8	7.09	6	5.39	14
B4	7.06	5	5.43	11	7.14	5	5.64	7
B5	8.06	1	5.26	13	8.11	1	5.51	12
B6	7.98	2	5.37	12	7.97	2	5.56	9
B7	5.01	14	6.43	1	4.95	14	6.22	1
B8	5.49	13	5.63	7	5.86	13	5.93	4
C1	6.26	11	5.97	3	6.35	12	6.01	3
C2	5.9	12	5.68	6	6.43	11	6.15	2

**Table 5:** Importance and satisfaction of The Moor street indicators.

**(Math.) factor**	**The Moor street**							
	**Local**				**Traveler**			
	**Importance**		**Satisfaction**		**Importance**		**Satisfaction**	
	**Mean**	**Ranking**	**Mean**	**Ranking**	**Mean**	**Ranking**	**Mean**	**Ranking**
A1	7.56	3	5.9	4	7.54	3	5.99	2
A2	7.3	4	5.58	10	7.1	5	5.7	11
A3	6.62	9	5.63	8	6.81	8	5.78	8
A4	6.5	10	5.95	3	6.67	10	5.83	6
A5	5.13	14	5.6	9	5.05	14	5.44	15
B1	7.15	7	5.04	15	6.8	9	5.47	14
B2	7.01	8	5.55	12	7.02	6	5.71	10
B3	7.23	5	5.49	14	6.94	7	5.56	12
B4	7.23	6	5.5	13	7.22	4	5.74	9
B5	8.32	1	5.58	11	8.02	1	5.81	7
B6	8.17	2	5.88	5	7.83	2	5.54	13
B7	5.02	15	6.41	1	4.8	15	5.95	4
B8	5.67	13	5.72	7	5.63	13	5.88	5
C1	6.49	11	6.13	2	6.23	11	6.01	1
C2	6.32	12	5.83	6	6.07	12	5.98	3

**Table 6:** Importance and satisfaction of West street indicators.

**(Math.) factor**	**West street**							
	**Local**				**Traveler**			
	**Importance**		**Satisfaction**		**Importance**		**Satisfaction**	
	**Mean**	**Ranking**	**Mean**	**Ranking**	**Mean**	**Ranking**	**Mean**	**Ranking**
A1	7.96	2	6.17	2	7.7	3	5.79	5
A2	7.72	4	5.51	8	7.43	4	5.63	8
A3	6.92	5	5.71	6	6.68	7	5.62	9
A4	6.79	6	5.99	4	6.77	5	5.73	6
A5	5.3	13	5.62	7	4.99	14	5.69	7
B1	6.43	9	4.9	15	6.41	10	4.89	15
B2	6.53	7	5.31	10	6.66	8	5.16	14
B3	6.27	11	5.24	11	6.44	9	5.37	13
B4	6.49	8	5.21	12	6.74	6	5.51	10
B5	8.06	1	5.03	14	7.9	1	5.42	12
B6	7.84	3	5.06	13	7.85	2	5.44	11
B7	5.04	14	6.13	3	4.85	15	6.08	2
B8	4.96	15	5.46	9	5.24	13	5.82	4
C1	6	12	5.87	5	6.25	12	6.04	3
C2	6.43	10	6.24	1	6.35	11	6.38	1

### 4.2 Extraction of implicit importance

**Figure 7:**
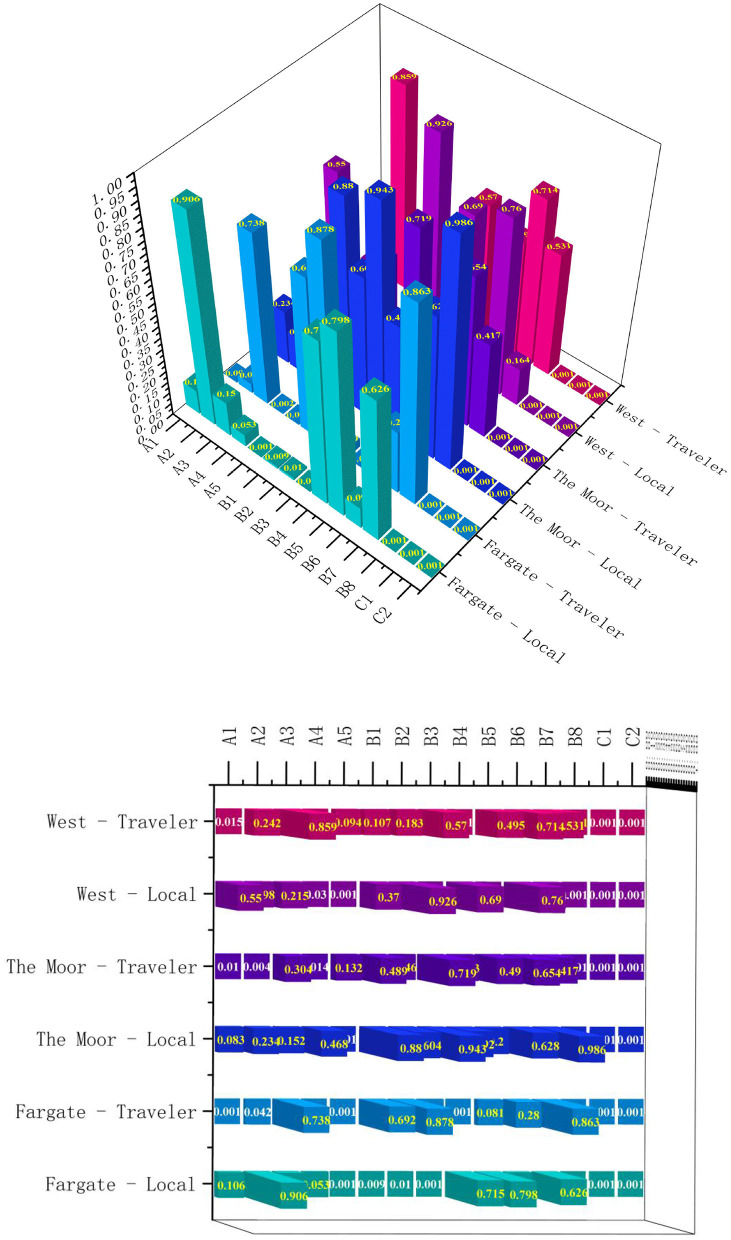
Significance statistics for Fargate, The Moor, WEST Street by locals and travelers.

**Figure 8:**
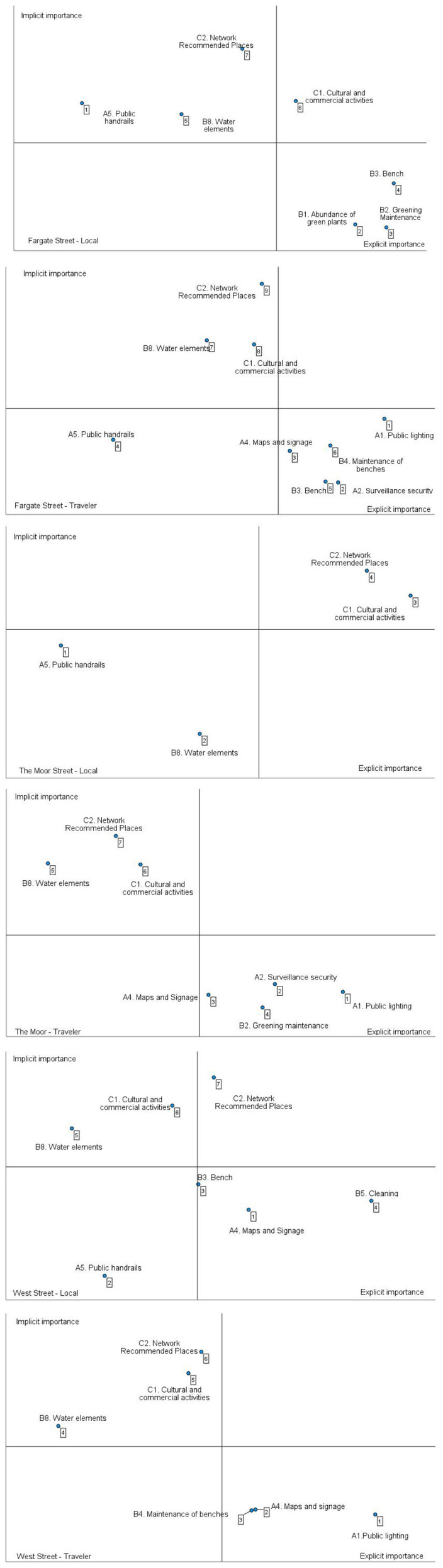
IPA-Kano quadrant analysis for three Sheffield streets under six scenarios.

Explicit importance is the direct evaluation of the importance of a street factor by the user, while implicit importance is the state of importance reflected in the user's evaluation of other aspects. Explicit and implicit are interrelated but not identical, so they must be compared and combined when assessing street factors. A *p* < 0.05 for street factors for the same user group is statistically significant. Whereas, factors with a *p* > 0.05 are not statistically significant (Figures 7, 8). According to the scores and rankings of implicit importance in Table 7, the implicit importance rankings of B8. Water element, C1. Cultural and commercial activities, and C2. Network Recommended Places are the same in all six cases, while the implicit importance rankings of the other elements are significantly different. It is worth noting that C2. Network Recommended Places ranked essentially first in all three streets.

**Table 7:** Implicit importance scores and ranking of the three street factors.

**(Math.) factor**	**Fargate Street**				**The Moor Street**				**West Street**			
	**Local**		**Traveler**		**Local**		**Traveler**		**Local**		**Traveler**	
	**Score**	**Ranking**	**Score**	**Ranking**	**Score**	**Ranking**	**Score**	**Ranking**	**Score**	**Ranking**	**Score**	**Ranking**
A1	0.136	10	**0.287**	**4**	0.142	5	**0.187**	**5**	0.048	11	**0.178**	**10**
A2	0.01	15	**0.144**	**9**	0.097	9	**0.208**	**4**	0.133	7	0.086	13
A3	0.121	11	0.024	13	0.117	7	0.154	7	0.1	9	0.013	15
A4	0.162	8	**0.215**	**7**	0.06	10	**0.179**	**6**	**0.174**	**6**	**0.189**	**8**
A5	**0.384**	**3**	**0.24**	**5**	**0.313**	**3**	0.11	9	**0.026**	**13**	0.123	11
B1	**0.219**	**6**	0.028	12	0.139	6	0.051	12	0.072	10	0.119	12
B2	**0.215**	**7**	0.011	15	0.043	12	**0.145**	**8**	0.007	15	0.98	1
B3	**0.275**	**5**	**0.146**	**8**	0.006	14	0.026	15	**0.231**	**4**	0.42	5
B4	0.031	13	**0.227**	**6**	0.056	11	0.102	10	0.032	12	**0.187**	**9**
B5	0.022	14	0.124	10	0.105	8	0.05	13	**0.194**	**5**	0.5	3
B6	0.141	9	0.077	11	0.04	13	0.033	14	0.025	14	0.27	7
B7	0.041	12	0.012	14	0.001	15	0.059	11	0.112	8	0.046	14
B8	**0.369**	**4**	**0.463**	**2**	**0.274**	**4**	**0.533**	**2**	**0.356**	**3**	**0.376**	**6**
C1	**0.387**	**2**	**0.454**	**3**	**0.335**	**2**	**0.53**	**3**	**0.407**	**2**	**0.494**	**4**
C2	**0.458**	**1**	**0.59**	**1**	**0.346**	**1**	**0.607**	**1**	**0.47**	**1**	**0.542**	**2**

The purpose of the bold is to reflect the similar rankings between elements that are the same on the three streets, to help in the analysis of the IPA-Kano model later.

### 4.3 IPA-Kano quadrant analysis

The evaluation results for the three street elements in the six scenarios have both similarities and differences (Figure 8, Table 8). The similarities are reflected in the fact that the same street factors belong to the same category of quadrants. Among the basic elements, two common points appear for all three streets, A1. Public lighting and A4. Maps and Signage. In terms of the importance factor, Fargate Street and The Moor Street both have the street factor C1. Cultural and commercial activities, while the street factor C2. Network Recommended Places is shared by The Moor Street and West Street. In terms of the attractiveness factor, B8. Water element, C2. Network Recommended Places and C1. Cultural and commercial activities are common to all three streets.

**Table 8:** Element evaluation results for Sheffield streets.

**Attributes of element**	**Fargate Street**		**The Moor Street**		**West Street**	
	**Local**	**Traveler**	**Local**	**Traveler**	**Local**	**Traveler**
Basic element	B1 B2 B3	A1 A2 A4 B3 B4		A1 A2 A4 B2	A4 B3 B5	A1 A4 B4
Important element	C1		C1 C2		C2	
Unimportant element		A5	A5 B8		A5	
Attractive element	A5 B8 C2	B8 C1 C2		B8 C1 C2	B8 C1	B8 C1 C2

The difference is that Fargate Street has more B1. Greenery abundance than the other two streets and West Street has more B5. Cleaning than the other two streets. The Moor Street has both C1. Cultural and commercial activities and C2. Network Recommended Places as important elements, whereas the other two streets only have one of these elements each. One of these Fargate Street has more A5. Public handrails than the other two streets in the attractiveness element.

### 4.4 Comparative analysis of evaluation results between locals and travelers

There is homogeneity in the assessment results between different streets, so we should focus on all these common elements (Table 9). From Table 9, it can be seen that the optimization paths of the three streets are mainly in the two indicators of safety and comfort, where A4. Maps and Signage are the priority to be improved in all three streets. In Fargate Street, locals consider the elements B1. Abundance of green plants and B2. Greening maintenance is important in the comfort indicator, whereas travelers consider A2. Surveillance security, A4. Maps and signage, B3. Benches, and B4. Maintenance of benches is important. Therefore, from the comparison of the two user groups of this street, the travelers would like to have more clean and well-maintained benches for resting while being safe, while the locals would like to see more different plants and timely care of the greenery, which would allow them to be more comfortable to move and live on this street. In The Moor Street, locals find the street satisfactory for the time being, while travelers think A4. Maps and Signage and B2. Greening maintenance is important. Therefore, this street could be prioritized for improvements based on the needs of travelers by improving the A4. Maps and Signage to make it easier and safer for travelers to move around the street and maintaining the greenery in a timely manner to make it more comfortable for travelers. In West Street, locals think that A4. Maps and Signage are important, travelers think that A1. Public lighting, A4. Maps and Signage, and B4. Maintenance of benches is important, and the addition of B8. Water elements can attract travelers better. Based on the comparison between locals and travelers, it is clear that West Street should prioritize the improvement of A4. Maps and Signage.

**Table 9:** Three street improvement priorities.

**Improvement priority**	**Fargate street**		**The Moor street**		**West street**	
	**Local**	**Traveler**	**Local**	**Traveler**	**Local**	**Traveler**
Priority 1						
Priority 2						
Priority 3						
Priority 4	B1 B2	A2 A4 B3 B4		A4 B2	A4	A1 A4 B4
Priority 5						
Priority 6						B8

In combining the concerns of the two user groups on the three streets, the safety indicators A2. Surveillance security, A4. Maps and Signage and A1. Public lighting elements should be prioritized for improvement, followed by the comfort indicators.

## 5 Discussion

### 5.1 Optimization pathways

According to the results of Table 9, Maps and Signage are the single most important factor according to both locals and travelers, Maintenance of benches and Greening maintenance are the second most important factor, while the remaining Abundance of green plants. Surveillance security, benches, public lighting, and water elements were ranked third in importance. Therefore, the final analysis of this study concludes that the local residents and travelers common concerns are Maps and Signage, maintenance of benches and Greening maintenance.

From the tourists' point of view, the study by Keliikoa et al. (41) confirms that maps with signage guidance are most useful for travelers who are new to the area. Ahmad Shafee et al. (42) argue that there is clear signage for tourists to refer to ensure the safety of the tourists. Literature and this study map and signage guidance have the same findings. And from the perspective of the locals, the study of Keliikoa et al. (41) concluded that the local occupants prefer to walk on their familiar routes. Mashary Alnaim et al. (17) based on the results of the study proposed to design the seats in the public space suitable for the location, increase the number of seats as well as increase the greenery and pay attention to its maintenance. Therefore, in comparing the results through the literature, the results of this study are scientifically valid.

Locals are more concerned about greenery a little more compared to travelers, while travelers are most concerned about maps and signage guidance. Therefore, the most popular interventions to address the locals' concerns about greenery are ecological interventions (43), which can enhance the sensory elements of the street by planting more different types of plants, and also, timing the maintenance of greenery by landscapers, this study will increase the awareness and understanding of policymakers about the preferences of the locals, to contribute to the increase of the life satisfaction, adoption of further improvements and adoption of future policies. For travelers, safety is the most important factor to consider, and according to the evidence from Ryan et al. (44) study, signage guidance for recreational walking can enhance psychological competence-perceived anxiety, confidence, safety, and route planning by addressing self-problems, therefore, in the process of designing maps and signage guidance, the needs of travelers are the most important factor. Process, the needs of the traveler are of importance and can be tailored to the traveler's route of travel, whilst ensuring that the signage is relevant to the local street.

### 5.2 Inadequate research

There are some limitations in the research process, for example, the lack of city-to-city comparisons, where the results obtained in one city can only see the problems of that city, but city-to-city comparisons can be analyzed in a more informative way with different people and environments. In addition, some of the indicators should be subdivided for the study, and the participants will be more specific in their evaluation of a particular element of the street. At the same time, IPA-Kano has some defects, the closer the factors are to the axis, the worse their attributes are. Because of the multicollinearity between the factors, binary correlation analysis was used to derive their implied importance instead of the usual partial correlation and regression analyses.

## 6 Conclusion

Using three streets in Sheffield, the IPA-Kano model was used to identify three categories of indicators of travelers and locals to the streets, resulting in basic, important and attractive elements, and by integrating these three categories of elements and their implicit importance, the locals were compared with travelers and an optimization path for the streets was established. The final result is that Maps and Signage are the most prioritized elements. The Greening maintenance and Maintenance of benches are a secondary issue, and by increasing the number of Maps and Signage, hiring a landscaper to take care of the greenery, and hiring a cleaner to maintain the benches, the safety and comfort of the street for both locals and travelers are met.

The IPA-Kano model used in this paper and its conclusions can help developers and designers to understand the current operating conditions and public preferences of their projects, as well as the key elements affecting street satisfaction, and provide data and science to support improvement and optimization for further projects.

## Data availability statement

The original contributions presented in the study are included in the article/supplementary material, further inquiries can be directed to the corresponding author.

## Author contributions

SH: Writing – original draft, Writing – review & editing.
